# Personality and gender prototypes for predicting health: a multilevel and cluster analysis

**DOI:** 10.1007/s00737-023-01385-2

**Published:** 2023-11-03

**Authors:** Sara Esteban-Gonzalo, Juan Carlos Fernández-Gonzalo, Juan Luis González-Pascual, Carmen Bouzas-Mosquera, Laura Esteban-Gonzalo

**Affiliations:** 1 Department of Social, Work and Differential Psychology, Faculty of Psychology, Universidad Complutense de Madrid, Campus de Somosaguas, Ctra. de Húmera, s/n 28223, Pozuelo de Alarcón, Madrid, Spain; 2 Faculty of Biomedicine, Nursing Department, Universidad Europea de Madrid, Calle Tajo, s/n 28670, Villaviciosa de Odón, Madrid, Spain; 3 Faculty of Physiotherapy and Nursing, Nursing Department, Universidad de Castilla-La Mancha, Avenida de Carlos III, s/n 45071, Toledo, Spain; 4 Faculty of Nursing, Physiotherapy and Podiatry, Nursing Department, Universidad Complutense de Madrid, Spain, Pl. de Ramón y Cajal, 28040 Madrid, Spain

**Keywords:** Personality, Gender, Prototypes, Health

## Abstract

Prior studies have identified that mentally healthy people tend to share common characteristics and common ways of coping with stressful life events; they show similar patterns of behavior and more flexible and adaptive social roles. The objectives of the study are to assess the influence of personality factors on gender roles and mental health, to identify personality patterns along with gender roles, and to assess the influence of the patterns identified on mental health. Data collection from a sample of 795 university students was carried out during 2019. Multilevel analyses tested the associations between gender (BSRI) and personality (TIPI) and between personality and health -mental health (GHQ12) and wellbeing (MHC-SF). Cluster analysis explored tendencies of gender and personality, and each cluster showed different health patterns. Individuals with high scores in extraversion, conscientiousness, emotional stability, openness to experience, and very high agreeableness score, as well as high femininity and masculinity scores, presented a decreased psychological morbidity (*β*= −3.62, 0.57 (SE), *p*<0.001) and an increased well-being (*β*=7.68, 1.15 (SE), *p*<0.001). The most relevant indicators of mental health were identified in androgynous individuals, those individuals with high scores in masculinity and femininity, as well as high scores in extraversion, openness to experience, emotional stability, agreeableness, and conscientiousness.

## Introduction

Biopsychosocial perspectives in health are indisputable; reciprocal influences of psychological, biological, and social forces act through intrapersonal, interpersonal, and contextual variables (Lehman et al. 2017). That some persons are more prone to disease and premature death is a corroborated fact given that certain individual differences could predispose to or protect against a great range of morbidity risk factors. Biopsychosocial characteristics of individuals and their variation over time, life decisions, lifestyles, and life priorities have an impact on health (Hampson and Friedman 2008).

Nowadays, it is known that disease patterns of individuals cannot be understood without taking into account other behavioral tendencies associated with cognitive styles, personality traits, and social roles (Bavolar and Bacikova-Sleskova 2018). For instance, it has been shown that impulse control is broadly related to a number of health behaviors, both positive behaviors that promote health, and unhealthy behaviors that put health at risk (Edmonds et al. 2009).

The link between some personality traits and processes of aging and illness has been consistently documented in different countries and cultures (Costa Jr et al. 2001). Particularly, associations between mental health and personality are supported by a robust body of research. Personality factors have been associated with coping strategies and resilience (Ferguson 2001), as well as with differential exposition to risk factors and circumstances in which mental morbidity would arise. Individual differences in personality traits may have important effects at the onset, development, and treatment of psychopathologies such as substance abuse, psychotic disorders, anxiety, and mood disorders (Fassino et al. 2013). A case in point, neuroticism and conscientiousness were associated with increased risk for Alzheimer’s, major depression, worse self-rated health, and overall greater disease burden (Turiano et al. 2012).

If we apply the same arguments used above, it would seem reasonable to believe that personality could also be associated with social roles (Lodi-Smith and Roberts 2007). Personality traits may influence motives, values, and abilities, interacting with people’s identities and thus conforming social roles (Lodi-Smith and Roberts 2007). The study of gender roles is one of the fields in which this relationship has become plausible. There is a robust body of research, substantiated cross-culturally, that links neuroticism and agreeableness with feminine gender roles and assertiveness and openness to experience with masculine gender roles. While being nice with others and being emotionally intense have been associated with the role of women in society, being assertive and open to try new things without fear and limitations are more connected to what is expected from men (Vianello et al. 2013).

Finally, based on the same associative principles, it would not be unreasonable to establish a relationship between social roles and health, as numerous studies have already suggested. In particular, it is now indisputable that there is a relationship between gender roles and health, (Hawkes and Buse 2013; King et al. 2020). From Sandra Bem’s models to the present, it is undeniable that certain gender roles may favor mental health, while others may deteriorate it (Bem 1985; del Pilar Sánchez-López et al., 2012). According to Sandra Bem, androgyny should be defined as the presence of both masculine and feminine traits in both men and women, and this condition has been associated to positive mental health outcomes (Bem 1985; Esteban-Gonzalo et al. 2021).

In an attempt to bring the previous ideas together, this study postulates that patterns of behaviors, feelings, thoughts, and motives shape human interactions and social roles, conforming different social identities (Weisberg et al. 2011). Behavioral tendencies, cognitive styles, and social roles may have an impact on health (Lehman et al. 2017; Woods 2019). Patterns of health and disease may be understood and probably should be understood, from a perspective in which health professionals are able to appreciate how certain ways of acting, feeling, believing, relating, and social positioning favor or hinder well-being and health. For example, the study of contextual variables related to gender such as exposure to caregiving strain or gender discrimination would be of great help in explaining and diagnosing certain health problems, more than sex itself (Nielsen et al. 2021).

Therefore, the aims of the present study are (1) to assess the influence of personality factors on gender roles (femininity/masculinity) and mental health (psychological morbidity and well-being); (2) to identify personality patterns along with gender roles; and (3) to assess the influence of the patterns identified on mental health of university students residing in Spain.

## Method

### Study design and participants

In the present cross-sectional study, participants were recruited through a convenience sampling from several Universities of Madrid and Toledo (Spain) and a variety of academic studies. The inclusion criteria were to be enrolled in any university course and to be able to fill in the questionnaire in the Spanish language. Once potential participants were informed of the study, all those university students who accepted to participate and gave their written consent were recruited. Data were collected over a university course period from February to November 2019. A total of 795 university students were included in the present study.

### Instruments

#### Personality

Personality was assessed using the Ten-Item Personality Inventory (TIPI), a brief personality scale based on the Big Five personality dimensions. Thus, separated scores for extraversion, agreeableness, conscientiousness, emotional stability, and openness to experience were obtained. Scores range from 1 to 7, 7 being the maximum expression of extraversion (as opposite to introversion), agreeableness (as opposite to disagreeableness), conscientiousness (as opposite to unconscientiousness), emotional stability (as opposite to neuroticism), and openness to experience (rejection of new experiences). The Spanish version was validated showing adequate psychometric properties. Test-retest correlations were strong, and convergence with the NEO-PI-R factors was significant (Renau et al. 2013).

#### Mental health

The General Health Questionnaire (GHQ-12), a commonly employed screening tool to identify individuals suffering psychological morbidity and symptomatology, was used to value psychological morbidity. The test consists of 12 items in which participants are asked to rate themselves on a 4-point Likert scale, indicating how often they had felt in a certain way during the last month. The test facilitates a total score in which higher scores indicate higher risk of mental morbidity. The Spanish version of the questionnaire was validated and showed internal consistency ranges between 0.82 and 0.90, sensitivity ranging between 76 and 100, and a reliability of 0.76 (Cronbach’s alpha). A higher score is associated with poorer mental health (del Pilar Sánchez-López and Dresch 2008; Muñoz et al. 1993).

In addition, the short form of the Mental Health Continuum (MHC-SF) was employed to assess emotional, psychological and social well-being. The MHC-SF consists of 14 items that measure emotional (3 items), social (5 items), and psychological well-being (6 items), facilitating a total score. Participants were asked to rate on a 6-point Likert scale how often they had felt a certain way over the last month. Higher scores indicate higher well-being (Keyes et al. 2008). The Spanish version of the MHC-SF was used which maintains the original structure. MHC-SF showed good reliability, Cronbach’s α (0.94) indicates an excellent internal consistency of scores from both the total scale and its subscales (Echeverría et al. 2017).

#### Gender roles

The Bem Sex-Role Inventory (BSRI) was used to value masculine and feminine gender roles separately, which includes perceptions, behaviors and attitudes. Thus, of the 60 items that make up the scale, 20 correspond to the assessment of masculinity and another 20 to that of femininity, and the rest are neutral items. The instrument provides two different independent scores for masculinity and femininity as well as a typology among the four available: masculine, feminine, undifferentiated and androgynous. The test consists of attributes with which subjects may feel more or less identified, such as “aggressive” and “analytical,” two examples of masculine-related traits.

The BSRI showed appropriate psychometric properties and is one of the most frequently employed validated gender role questionnaires across countries and age groups. In addition, it has been specifically utilized to study the relationship between gender and health (Spence et al. 1974; Vafaei et al. 2014). For the present study, the Spanish version of the BSRI was employed, respecting the original structure, with adequate reliability and Cronbach’s alpha of 0.77 and 0.78 for both subscales, as has recently been validated (Vafaei et al. 2014).

#### Covariates

Several covariates were included in the analysis performed with the objective of controlling the confounding effect of those variables with potential influence on personality, gender roles and mental health. These covariates were age, socioeconomic status (SES) and sex.

### Ethical considerations

The Ethics Committee of the Faculty of Biomedical and Health Science of the Universidad Europea de Madrid approved the study protocol.

### Data analyses

Multilevel mixed-effects linear regression was employed to determine (1) the association between personality factors and femininity/masculinity and (2) the association between personality factors and mental health (psychological morbidity and well-being) (Table 2). This instrument was used in order to address levels of grouping present among the participants examined. All models included a random intercept for university, academic field, and class group. Non-normal variables were transformed to address normality using a logarithmic function in all cases. Unadjusted models and models adjusted for age, SES, and reported sex were fitted. Each covariate was included one by one in the models for each analysis performed (data not shown due to the lack of effect of the covariates).

In addition, cluster analysis was carried out in order to detect relatively homogeneous groups with respect to patterns of personality and femininity/masculinity. Hierarchical clustering using Ward’s method was carried out considering standardized scores and Squared Euclidean distances. Cluster consistency was assessed employing Kruskal-Wallis and Chi square test (Table 3). Finally, additional multilevel mixed-effects linear regression models were calculated to value the influence of personality and gender role clusters and mental health (Table 4), following the same statistical protocol previously mentioned.

## Results

Characteristics of the sample are presented in Table 1. Statistically significant results will be described exclusively in this paragraph, even with discrete correlation coefficient values. Higher scores of extraversion and openness to experience were both associated with higher masculinity (*r*=0.32, *p*<0.001; *r*=0.25, *p*<0.001, respectively) and femininity scores (*r*=0.07, *p*=0.048; *r*=0.11, *p*=0.002), as well as with lower psychological morbidity scores (*r*= −0.19, *p*<0.001; *r*= −0.09, *p*=0.009), higher well-being scores (*r*=0.26, *p*<0.001; and *r*=0.08, *p*=0.018), and higher SES (*r*=0.07, *p*=0.048; *r*=0.09, *p*=0.016). A higher score for agreeableness was related to lower masculinity scores (*r*= −0.14, *p*<0.001), higher femininity scores (*r*=0.38, *p*<0.001), lower psychological morbidity scores (*r*= -0.08, *p*=0.015), higher well-being scores (*r*=0.12, *p*=0.001), and higher age (*r*=0.09, *p*=0.012). Finally, higher scores for conscientiousness and emotional stability were both linked to higher masculinity (*p*<0.001, *r*=0.14; *p*=0.041, *r*=0.07, respectively) and femininity scores (*r*=0.17, *p*<0.001; *r*=0.15, *p*<0.001), as well as to lower psychological morbidity scores (*r*= −0.08, *p*=0.017; *r*= -0.37, *p*<0.001), greater well-being scores (*r*=0.22, *p*<0.001; *r*=0.33, *p*<0.001), higher age (*r*=0.07, *p*=0.033; *r*=0.12, *p*=0.001), and sex (both *p*<0.05). Female subjects showed a higher average of conscientiousness, while male subjects showed a higher average of emotional stability.

**Table 1:** Characteristics of the participants examined (*n*=795)

	Participants examined^a^	Extraversion	Agreeableness	Conscientiousness	Emotional stability	Openness to experience
*n*	795					
Personality factors						
Extraversion (1–7)	4.6 (1.4)	–	–	–	–	–
Agreeableness (1–7)	5.4 (1.0)	–	–	–	–	–
Conscientiousness (1–7)	5.1 (1.1)	–	–	–	–	–
Emotional stability (1–7)	4.5 (1.3)	–	–	–	–	–
Openness to experience (1–7)	5.2 (1.1)	–	–	–	–	–
Masculinity (10–30)	4.7 (0.6)	**<0.001 (0.32)**	**<0.001 (−0.14)**	**<0.001 (0.14)**	**0.041 (0.07)**	**<0.001 (0.25)**
Femininity (6–36)	4.9 (0.6)	**0.048 (0.07)**	**<0.001 (0.38)**	**<0.001 (0.17)**	**<0.001 (0.15)**	**0.002 (0.11)**
Psychological morbidity score (12–48)	23.8 (5.9)	**<0.001 (−0.19)**	**0.015 (−0.08)**	**0.017 (−0.08)**	**<0.001 (−0.37)**	**0.009 (−0.09)**
Well–being score (14–84)	60.8 (12.5)	**<0.001 (0.26)**	**0.001 (0.12)**	**<0.001 (0.22)**	**<0.001 (0.33)**	**0.018 (0.08)**
Socioeconomic status (0–5)	4.0 (1.0)	**0.048 (0.07)**	0.104 (0.06)	0.621 (**−**0.01)	0.126 (0.05)	**0.016 (0.09)**
Age	22.5 (5.6)	0.306 (0.03)	**0.012 (0.09)**	**0.033 (0.07)**	**0.001 (0.12)**	0.155 (**−**0.05)
Sex		0.217	0.312	**<0.001**	**0.007**	0.403
Female	69.6 (553)^b^	4.57 (1.42)^a^	5.37 (1.02) ^a^	5.26 (1.17) ^a^	4.42 (1.33) ^a^	5.25 (1.17) ^a^
Male	30.4 (242)^b^	4.69 (1.47) ^a^	5.43 (1.04) ^a^	4.82 (1.13) ^a^	4.85 (1.25) ^a^	5.11 (1.16) ^a^

^a^Values are mean (SD)

^b^Values are percentage (number). Spearman’s correlation test, *p* (correlation coefficient), except comparison between sex and personality factor Kruskal-Wallis test; *p* statistical values are shown in bold

In addition, negative correlation was detected between psychological morbidity and well-being (*p* <0.001, *r*= −0.50) (data not shown in table). The higher the well-being, the lower the psychological morbidity.

Linear mix models for the associations between personality factors and femininity/masculinity and between personality factors and psychological morbidity and well-being are shown in Table 2. Since no relevant differences were detected with respect to results found in the unadjusted models, only those results associated with the adjusted models are shown, with two exceptions. A one-unit increase in extraversion scores was related to higher masculinity (*β*=0.13, 0.01(SE), *p*<0.001) and well-being scores (*β*=2.27, 0.33 (SE), *p* <0.001), as well as with a lower psychological morbidity score (*β*= −0.82, 0.16 (SE), *p* <0.001). A one-unit increase in agreeableness was related to increased femininity (β=0.23, 0.02(SE), *p*<0.001) and well-being scores (*β*=0.10, 0.45 (SE), *p*=0.016) (the latter only in the unadjusted model, with the differences disappearing in the adjusted model) and with a decreased masculinity score (*β*= −0.12, 0.02 (SE), *p*<0.001). A one-unit increase in conscientiousness was associated with greater masculinity (*β*=0.11, 0.02 (SE), *p*<0.001), femininity (*β*=0.07, 0.02 (SE), *p*=0.002), and well-being scores (*β*=2.08, 0.41 (SE), *p*<0.001), as well as with a decreased psychological morbidity score (*β*= −0.48, 0.20 (SE), *p*=0.015). A one-unit increase in emotional stability was linked to increased femininity (*β*=0.09, 0.02 (SE), *p*<0.001), well-being scores (*β*=3.35, 0.35 (SE), *p*<0.001), and a decreased psychological morbidity score (*β*= −1.72, 0.17 (SE), *p*<0.001). Finally, a one-unit increase in open to experience was associated with an increased masculinity score (*β*=0.14, 0.02 (SE), *p*<0.001) and a decreased psychological morbidity score (*β*= −0.46, 0.20 (SE), *p*=0.025).

**Table 2:** Linear mix models for the association between personality factors and femininity/masculinity, psychological morbidity, and well-being

	Unadjusted model				Adjusted model			
	*n*	*b* (SE)	95% CI	*p*	*n*	*b* (SE)	95% CI	*p*
*Extraversion*								
Masculinity score	601	0.14 (0.01)	0.10–0.17	**<0.001**	552	0.13 (0.01)	0.10–0.17	**<0.001**
Femininity score	621	0.03 (0.01)	−0.00–0.06	0.066	567	0.03 (0.01)	−0.00–0.07	0.050
Psychological morbidity	672	−0.84 (0.15)	−1.14 to −0.53	**<0.001**	616	−0.82 (0.16)	−1.14 to −0.51	**<0.001**
Well-being score	682	2.28 (0.31)	1.66–2.90	**<0.001**	624	2.27 (0.33)	1.62–2.92	**<0.001**
*Agreeableness*								
Masculinity score	600	−0.12 (0.02)	−0.17 to −0.07	**<0.001**	553	−0.12 (0.02)	−0.18 to −0.07	**<0.001**
Femininity score	621	0.22 (0.02)	0.18–0.27	**<0.001**	568	0.23 (0.02)	0.18–0.28	**<0.001**
Psychological morbidity	671	−0.40 (0.22)	−0.84–0.02	0.066	617	−0.39 (0.23)	−0.85–0.06	0.095
Well-being score	682	1.10 (0.45)	0.20–1.99	**0.016**	626	0.90 (0.48)	−0.05–1.84	0.065
*Conscientiousness*								
Masculinity score	599	0.07 (0.02)	0.03–0.12	**0.001**	550	0.11 (0.02)	0.06–0.15	**<0.001**
Femininity score	618	0.08 (0.02)	0.04–0.13	**<0.001**	565	0.07 (0.02)	0.02–0.11	**0.002**
Psychological morbidity	670	−0.32 (0.19)	−0.69–0.05	0.096	615	−0.48 (0.20)	−0.87 to −0.09	**0.015**
Well-being score	680	2.03 (0.38)	1.27–2.79	**<0.001**	623	2.08 (0.41)	1.28–2.89	**<0.001**
*Emotional stability*								
Masculinity score	600	0.03 (0.02)	−0.00–0.07	0.079	552	0.02 (0.02)	−0.01–0.06	0.197
Femininity score	620	0.08 (0.01)	0.04–0.11	**<0.001**	567	0.09 (0.02)	0.05–0.13	**<0.001**
Psychological morbidity	670	−1.78 (0.16)	−2.10 to −1.46	**<0.001**	615	−1.72 (0.17)	−2.06 to −1.38	**<0.001**
Well-being score	680	3.24 (0.33)	2.58–3.90	**<0.001**	623	3.35 (0.35)	2.65–4.05	**<0.001**
*Openness to experience*								
Masculinity score	601	0.13 (0.02)	0.09–0.17	**<0.001**	552	0.14 (0.02)	0.10–0.19	**<0.001**
Femininity score	621	0.04 (0.02)	−0.00–0.08	0.057	567	0.02 (0.02)	−0.02–0.06	0.368
Psychological morbidity	672	−0.43 (0.19)	−0.82 to −0.04	0.028	616	−0.46 (0.20)	−0.86 to −0.05	**0.025**
Well-being score	682	0.56 (0.40)	−0.22–1.36	0.162	624	0.53 (0.42)	−0.30–1.37	0.210

Statically significant values are in bold. Adjusted model: analyses were adjusted for age, socioeconomic status, and reported sex*.* All b-values are unstandardized coefficients and represent the increase in mental health indicators with a one-unit increase in the predictor

Clustering of personality factors along with gender roles is presented in Table 3. Subsequent analysis identified three patterns of personality and masculinity and femininity as the most suitable and homogenous groups of individuals, showing significant differences among each other.

**Table 3:** Clustering of personality factors and gender roles

*N* 623 *N* (males), 202 *N* (females), 421	Group 1 (*n*=63) (*n*=113)	Group 2 (*n*=73) (*n*=192)	Group 3 (*n*=66) (*n*=116)	*p* 0.908**
Extraversion	5.28 (1.18)+	3.63 (1.27)-	5.25 (1.14)+	<0.001*
Agreeableness	6.19 (0.62)+	5.65 (0.80)+	4.33 (0.81)-	<0.001*
Conscientiousness	5.81 (0.86)+	4.98 (1.12)	4.80 (1.09)	<0.001*
Emotional stability	5.60 (1.01)+	4.35 (1.25)-	4.02 (1.15)-	<0.001*
Openness to experience	5.92 (0.80)+	4.81 (1.10)	5.18 (1.25)+	<0.001*
Masculinity	5.07 (0.56)+	4.36 (0.58)-	5.12 (0.52)+	<0.001*
Femininity	5.37 (0.57)+	4.89 (0.64)	4.74 (0.62)	<0.001*

Values are mean (SD)

Significant *p* values in bold

*Cluster 1* androgynous group, *Cluster 2* feminine group, *Cluster 3* masculine group

*Kruskal-Wallis test; **Pearson chi-square test

^+^Above the average

^-^Below the average

Cluster 1 (the androgynous group), made up of 176 participants, was characterized by high scores of extraversion, conscientiousness, emotional stability, openness to experience, and a very high agreeableness score, as well as high femininity and masculinity scores. Cluster 2 (the feminine group) consisted of 265 participants who registered low extraversion and emotional stability scores, high agreeableness, and medium-high conscientiousness scores, as well as low masculinity and medium-high femininity scores. Finally, cluster 3 (the masculine group) with 182 participants showed high extraversion, low agreeableness, medium conscientiousness, low emotional stability, and high openness to experience scores, as well as high masculinity and average femininity scores. *p* <0.001 for all cases.

Linear mix models for the association between personality and gender role clusters and psychological morbidity and well-being are shown in Table 4. Belonging to cluster 1 (the androgynous group) was associated with a decreased psychological morbidity score (*β*= −3.62, 0.57 (SE), *p*<0.001) and an increased well-being score (*β*=7.68, 1.15 (SE), *p*<0.001). Belonging to cluster 2 (the feminine group) was linked to an increased psychological morbidity score (*β*=2.98, 0.53 (SE), *p*<0.001) and a decreased well-being score (*β*= −7.32, 1.07 (SE), *p*<0.001).

**Table 4:** Linear mix models for the association between personality and gender roles clusters and psychological morbidity and well-being

	Unadjusted model				Adjusted model			
	*n*	*b* (SE)	95% CI	*p*	*n*	*b* (SE)	95% CI	*p*
*Psychological morbidity*								
Cluster 1	539	−3.48 (0.55)	−4.57 to −2.40	**<0.001**	496	−3.62 (0.57)	−4.75 to −2.50	**<0.001**
Cluster 2	539	2.88 (0.51)	1.87–3.90	**<0.001**	496	2.98 (0.53)	1.92–4.04	**<0.001**
Cluster 3	539	0.12 (0.57)	−0.99–1.25	0.824	496	0.20 (0.59)	−0.96–1.37	0.730
*Well-being*								
Cluster 1	542	7.45 (1.11)	5.26–9.64	**<0.001**	498	7.68 (1.15)	5.40–9.95	**<0.001**
Cluster 2	542	−7.04 (1.03)	−9.07 to −5.02	**<0.001**	498	−7.32 (1.07)	−9.43 to −5.20	**<0.001**
Cluster 3	542	0.75 (1.16)	−1.52–3.03	0.517	498	0.74 (1.21)	−1.63–3.11	0.541

Statically significant values are in bold. Adjusted model: analyses were adjusted for age, socioeconomic status, and reported sex. All *b*-values are unstandardized coefficients and represent the increase in mental health indicators with a one-unit increase in the predictor

*Cluster 1* androgynous group, *Cluster 2* feminine group, *Cluster 3* masculine group

## Discussion

The associations between personality traits and gender point in the same direction as those found by other authors. Masculinity is positively linked to extraversion, openness to experience and responsibility, while femininity is positively linked to agreeableness and also to conscientiousness and emotional stability.

The link between extraversion and openness to experience and masculinity has been documented by other studies (Costa Jr et al. 2001; Vianello et al. 2013). Considering that dominance, assertiveness, and agency have been characteristics traditionally associated with male gender role (Bem 1974; Mahalik et al. 2003), such traits appear to be consistent with both openness to new ideas and experiences (openness to experience) and extraversion (Costa Jr et al. 2001). It is known that several facets of extraversion have opposing associations with fear responses (Pineles et al. 2009). In fact, it has been found that extroverted individuals may have different learning processes, better process appetitive stimuli and generate responses of curiosity, desire, and euphoria to a greater extent (Carver and White 1994). Also, it has been found that extroverted individuals tend to have less fear of negative evaluation (Keighin et al. 2009). At the opposite pole, introverted individuals may tend to process aversive stimuli better, generating more anxiety and fear responses (Carver and White 1994).

Regarding femininity, the results were as expected for agreeableness, but not for emotional stability. Numerous studies have found a positive relationship between femininity and agreeableness (Costa Jr et al. 2001), which is not surprising considering that the female gender role has traditionally been associated with empathy and caring for others (Bem 1985; Mahalik et al. 2005). It has been hypothesized that communal-expressive traits (sharing of thoughts or feelings, caring) may be related to the typical demands of feminine gender role such as family roles like involvement in romantic relationships and the wish for children and parenthood (Abele 2003; Twenge 2001). However, the fact that femininity was positively associated with mental stability had not previously been found, as most studies have reported a positive correlation between femininity and neuroticism (Costa Jr et al. 2001). As we will see in later analyses, mental emotional stability is playing an ambiguous role in the results of this research, being associated with both female and male contexts. Finally, despite the fact that previous studies are not congruent regarding conscientiousness (Mitchell 1987), this trait was found to be associated with both masculinity and femininity in the present study, perhaps postulating as an androgynous trait.

Associations between personality traits and psychological health are aligned with those suggested by other researchers. Psychological morbidity was negatively linked to extraversion, openness to experience and emotional stability, while well-being was positively linked to agreeableness, conscientiousness, emotional stability, and extraversion.

These results are consistently supported by similar studies in the field. Emotional stability and extraversion have been found to prevent mental pathologies in several studies and different cultural contexts (Lamers et al. 2012; Ulu and Tezer 2010). They have been found to be a protective factor against depression, anxiety disorders, schizophrenia, eating disorders, and personality disorders (Cuijpers et al. 2010). In addition, openness to experience has also been found to be a protective factor against mental morbidity in the present study, which may be understood in terms of preventing depression (Carrillo et al. 2001) as well as positively regulating stress (Williams et al. 2009).

Previous researchers have also found well-being associated with agreeableness, emotional stability, and extraversion (Lamers et al. 2012; Schmitt et al. 2007). Some authors have suggested that positive mental health (the presence of feelings of well-being rather than just the absence of psychopathology) is positively associated with agreeableness and extraversion (Lamers et al. 2012). Similarly, while emotional stability seems to be more connected with feelings of positive affect, agreeableness and extraversion may be more linked to experiencing positive life events, engaging in more successful social situations such as positive relationships and community involvement (Lamers et al. 2012). Lastly, although the mechanisms of conscientiousness are less known, this trait appears to act as a good mediator between perceived control and all the previous aspects of well-being (Smith et al. 2013).

### Personality and gender typologies

The three personality and gender typologies seem congruent with the results previously discussed. Group 1, the androgynous group, shows high scores in both femininity and masculinity and thus seems to identify with the androgynous prototype defined by Sandra Bem (Bem 1985). Composed of adaptive, versatile and mentally healthy individuals, Group 1 shows high scores in extraversion, conscientiousness, emotional stability and openness to experience, as well as a very high agreeableness score. This connection also appears to be consistent with the androgyny model, according to which androgynous subjects show a greater capacity to adapt to new contexts and handle difficult situations (Bem 1985). Other authors have confirmed this link with androgyny’s positive effects on mental health (Esteban-Gonzalo et al. 2021; Lefkowitz and Zeldow 2006; Prakash et al. 2010). Additionally, our results showed that these individuals tend to be extroverts and emotionally stable, they enjoy trying new ideas and experiences, they tend to follow socially prescribed norms with goal orientation and planning (conscientiousness), and they are apt to be prosocial, gentle, flexible, and patient with others (agreeableness). According to the data obtained, the conditions of this group of people may provide them with better mental health, not only with a lower tendency to psychological morbidity, but also with greater well-being.

Group 2, the feminine group, also shows consistency with the previous analysis. The feminine group is characterized by low scores in extraversion and emotional stability, but high scores in agreeableness. This group also shows the most pessimistic scores in terms of mental health. The personality traits that accompany this prototype show a tendency toward introversion and emotional instability, both associated with psychological morbidity (Jylhä et al. 2009; Lahey 2009; Yen and Siegler 2003). Moreover, high scores in agreeableness (prosocial, gentle, flexible, and patient with others) are consistent with feminine prototype expectations (Abele 2003; Costa Jr et al. 2001).

Finally, group 3 (the masculine group) is defined by high scores in extraversion and openness to experience low agreeableness and low emotional stability and is neutral in terms of mental health indicators. Individuals in the masculine cluster are defined as extroverts, open to new ideas and experiences, with low tendency to behave in a prosocial, gentle and flexible way (agreeableness), which is congruent with previous studies on personality (Costa Jr et al. 2001). These results are also congruent with gender studies where it is observed that typically masculine traits are defined as instrumental traits (active, energetic, fearless, willing to make decisions, etc.), traits that are closely linked and connected with extraversion and openness to experience, and potentially better connected with health that expressive traits (typically feminine traits) (Bem, 1974, Bem, 1985). However, the low scores in emotional stability when compared with the androgenous cluster are striking. Although emotional stability has been a trait traditionally associated with male gender, this cannot be observed in our results as this male group presents the lowest scores in emotional stability. In fact, in this study it has played an ambiguous role, applicable to both male and female prototypes. Thus, emotional stability could act more as an androgynous trait, rather than masculine or feminine.

The study has some limitations that must be considered when interpreting the results. The cross-sectional nature of the data cannot infer causation; we can only report associations between mental health indicators and social, demographic, and economic factors. Perhaps unmeasured covariates or measurement error in the covariates included in the models could lead to residual confounding. The sample considered in this study is not representative of the general Spanish population. Thus, the results of the present study cannot be generalizable. Finally, future longitudinal studies should be carried out to extend the cross-sectional perspective examined in this study. Also, given the relationship between instrumental/expressive traits with some personality factors, future studies should replicate the study using other gender measures (roles, identities, etc.) and not only trait-based gender measures.

## Conclusions

The results of the present study allow us to assume that people with better mental health share a series of personality and gender-related traits that provide them with better tools for coping with the challenges they face. According to the data obtained, the best indicators of mental health have been identified in androgynous individuals with high scores in both masculinity and femininity, as well as high scores in extraversion, openness to experience, emotional stability, agreeableness, and conscientiousness. These results reinforce the biopsychosocial model of health according to which health professionals must be provided with tools that enable them to understand how people act, think, and adapt to their social environment in terms of personality and gender-related traits, in order to prevent and intervene in health problems.
